# Chaperone-Mediated Autophagy Regulates Hypoxic Pathology in Cardiomyocytes

**DOI:** 10.1080/27694127.2023.2174337

**Published:** 2023-02-07

**Authors:** Rajeshwary Ghosh, J. Scott Pattison

**Affiliations:** a Department of Nutrition and Integrative Physiology, Molecular Medicine Program, University of Utah, 15N 2030E, Salt Lake City, Utah 84112;; b Division of Basic Biomedical Sciences, Sanford School of Medicine, University of South Dakota, Vermillion, SD 57069, USA

**Keywords:** Cardiomyocytes, cell death, chaperone-mediated autophagy, hypoxia, ischemic heart disease, LAMP2A, lysosome

## Abstract

Tight regulation of protein degradation pathways is essential for maintaining cardiac homeostasis. The goal of this work was to define the role of chaperone-mediated autophagy (CMA), in cardiomyocytes. CMA acts as a selective degradation pathway of proteins using a cytosolic and lysosomal co-chaperone, HSPA8/HSC70, and the CMA-specific LAMP2A (lysosomal-associated membrane protein 2A) receptor. LAMP2A protein levels are known to be necessary for CMA function. While CMA was shown to exert protection against neurodegenerative disorders and cancer, the role of CMA during cardiac pathology was not known. It was hypothesized that enhancing CMA could mitigate hypoxic pathology in cardiomyocytes. Thus, a genetic gain- and loss-of-CMA-function approach was employed using a *Lamp2a*-overexpressing adenovirus and a *Lamp2a*-silencing siRNA, respectively, in primary cardiomyocytes treated with CoCl_2_ (a hypoxia-mimetic agent) or vehicle control. The experiments performed clearly showed that *Lamp2a*-overexpression leads to CMA activation that is sufficient to attenuate hypoxia-induced cardiomyocyte death and toxicity.

## CMA is active in the heart and modulated by hypoxic stress

Impaired protein degradation pathways typically result in the accumulation of disease-causing proteins, which contribute to the pathological progression of heart diseases. There are two major intracellular protein degradation pathways: the ubiquitin-proteasome system (UPS) and the macroautophagy-lysosome pathway, both of which participate in bulk protein degradation. While stimulating macroautophagy or the UPS have been examined as potential therapeutic avenues to treat cardiac pathology, clinical translation of these interventions is still lacking. The process of CMA is unique to mammalian cells and highly selective in terms of which proteins are sub-strates for this mechanism of degradation. However, whether CMA was functional in the heart was largely unknown.

The most common form of cardiac pathology is ischemic heart disease, which is preceded by myocardial infarction and characterized by tissue hypoxia. The study revealed several novel findings pertaining to the causal role of CMA in hypoxia-induced cardiomyocyte pathology [1]. Additionally, several pieces of experimental evidence demonstrated that CMA is functional in cardiomyocytes and is regulated by extrinsic stresses i.e., ischemia/hypoxia. Endogenous LAMP2A protein levels are significantly upregulated in hypoxically-stressed cardiomyocytes. Ischemic human myocardium shows an increase in LAMP2A protein levels (+1.7-fold) suggesting that CMA is activated during ischemic cardiac stress. The findings suggested that while cardiac CMA is upregulated in response to ischemic stress, the extent of CMA activation is insufficient to protect the heart against the ischemic pathology.

Several methods were employed to validate changes in LAMP2A and CMA activity in cardiomyocytes. In addition to measuring the total LAMP2A protein levels, LAMP2A levels were determined in intact lysosomal fractions (an optimal method for measuring CMA function). The intact lysosomal fractions have markedly increased LAMP2A-positive lysosome levels during hypoxic stress, suggesting that hypoxia upregulates CMA by increasing the LAMP2A-positive lysosomal content. Additionally, a bona fide CMA substrate, KFERQ-luciferase reporter construct, was designed to determine the changes in CMA activity in hypoxically-stressed cardiac cells. The CMA reporter assay validated that LAMP2A protein levels are directly correlated with CMA activity in the cells and treatments tested.

The study further confirmed MEF2D, a myocyte-specific transcription factor, as an endogenous substrate of CMA in cardiomyocytes. MEF2D protein levels are decreased with *Lamp2a*-overexpression and increased in the *Lamp2a*-silenced cardiomyocytes providing further evidence of CMA’s degradative function in cardiomyocytes. Determining the CMA-specific degradation of other functionally important cardiac and disease-causing proteins in intact lysosomes would further validate the significance of CMA function in the heart and advance the field of protein quality control in cardiac disease.

## Upregulating CMA reduces hypoxia-induced cardiomyocyte toxicity and cell-death

A pivotal finding of the study was that activating CMA protects the cardiomyocytes against hypoxia-induced cytotoxicity and apoptosis. CoCl_2_-induced hypoxia significantly increases apoptotic cell death, plasma membrane integrity loss, and cytotoxicity in primary cardiomyocytes. Intriguingly, the data demonstrated that gain of CMA function by *Lamp2a*-overexpression significantly reduces CoCl_2_-induced cell death and cytotoxicity, whereas loss of CMA function by *Lamp2a*-knockdown is detrimental. Apart from genetically-driven alterations of CMA, pharmacological agents including the HSP90 inhibitor geldanamycin (GA), and the PI3-kinase and PtdIns3K inhibitor, 3-methyladenine (3-MA), were utilized to gain insights on whether and how these agents regulated CMA. While GA aggravates cytotoxicity and is associated with decreased LAMP2A levels, 3-MA increases LAMP2A levels in cardiomyocytes and exhibits decreased cytotoxicity. Furthermore, the data showed that *Lamp2a*-silencing exacerbates, and *Lamp2a*-overexpression decreases cell death, and unveiled CMA activation as a novel potential mechanism to preserve cardiomyocyte viability and function.

## Upregulated CMA modestly increases macroautophagic flux

Another important finding of the study is that CMA activation through *Lamp2a*-overexpression causes a significant increase in macroautophagic activity/flux suggesting a form of crosstalk from CMA to macroautophagy exists. Although CMA and macroautophagy function independently in terms of their mechanisms of action, a cross-regulation between these two proteolytic pathways in mouse fibroblasts was previously shown. When LAMP2A is increased by two-fold, the enhanced CMA significantly increases macroautophagic flux confirming the existence of regulation across the two autophagic pathways. Additional studies are needed to further explore the mechanistic interaction between CMA, macroautophagy, and the UPS, to determine whether and how these pathways complement, interact, or antagonize one another in the heart.

## Conclusion

Studies related to mechanisms of protein degradation in the heart are rapidly progressing with the continuous discovery of novel therapeutic tools and targets. The present study demonstrated for the first time a protective role of CMA in cardiomyocytes against hypoxic stress and revealed the potential of targeting CMA activation for the treatment of ischemic heart disease. However, further mechanistic studies including the interaction of various disease-causing proteins with the CMA machinery and their turnover via CMA, in stressed hearts are required. Also, the effects of gain and loss of CMA using genetically-modified animals in heart failure pathology must be tested to determine the therapeutic significance of myocardial CMA. Targeting CMA degradation may uncover new avenues for the treatment of heart failure patients.
